# Molecular identification and diversity of adult arthropod carrion community collected from pig and sheep carcasses within the same locality during different stages of decomposition in the KwaZulu-Natal province of South Africa

**DOI:** 10.7717/peerj.12500

**Published:** 2021-11-29

**Authors:** Danisile Tembe, Mokgadi Pulane Malatji, Samson Mukaratirwa

**Affiliations:** 1 School of Life Sciences, College of Agriculture, Engineering and Science, University of KwaZulu-Natal, Durban, South Africa; 2 Foundational Research and Services, South African National Biodiversity Institute, Pretoria, South Africa; 3 One Health Center for Zoonoses and Tropical Veterinary Medicine, Ross University School of Veterinary Medicine, Basseterre, West Indies

**Keywords:** Forensic sciences, Forensic entomology, Arthropods diversity, Beetles, Flies, Cold season, Warm season

## Abstract

The current study aimed at molecular identification and comparing the diversity of arthropods communities between pig and sheep carcasses during the cold and warm season in KwaZulu-Natal province of South Africa. Adult arthropods found on and around the carcasses were collected using either fly traps or forceps. Molecular analyses confirmed the identification of twelve arthropod species collected from both sheep and pig carcasses during the cold season. Results showed that 11 of 12 arthropod species were common in both sheep and pig carcasses, with exception to *Onthophagus vacca* (Coleoptera: Scarabaeidae) (Linnaeus, 1767) and *Atherigona soccata* (Diptera: Muscidae) (Rondani, 1871) species which were unique to sheep and pig carcasses respectively. However, during the warm season, the sheep carcass attracted more arthropod (*n* = 13) species as compared to the pig carcass. The difference in the obtained arthropod was due to the presence of *O. vacca* which was also unique to the sheep carcass during this season. Furthermore, there was an addition of a beetle species *Hycleus lunatus* (Coleoptera: Meloidae) (Pallas, 1782), which was collected from both sheep and pig carcasses but unique to the warm season. The pig carcass attracted more dipteran flies during both warm (*n* = 1,519) and cold season (*n* = 779) as compared to sheep carcass during the warm (*n* = 511) and cold season (*n* = 229). In contrast, coleopterans were more abundant on the sheep carcass during the warm season (*n* = 391) and cold season (*n* = 135) as compared to the pig carcass in both warm season (*n* = 261) and cold season (*n* = 114). In overall, more flies and beetles were collected on both sheep and pig carcasses during the warm season, and this further highlight that temperature influenced the observed difference in the abundance of collected arthropod between seasons.

## Introduction

Arthropods are some of the major contributors to the community structure and ecosystems due to their high diversities, high densities which may result from their high reproductive rates, as well as their ability to occupy several trophic categories within communities (Azwandi et al., 2013). According to Castner (2001), insects are the largest groups of phylum Arthropoda with the most numerous and diverse species on the planet. There are four categories of insects species that can be found on a decomposing carcass: first, necrophagous species which use the carcass for feeding or oviposition, followed by the predators which are insects feeding on other insects or arthropods as a food source, the omnivores such as ants, beetles and wasps, which feed on the carcass and its colonizers, and lastly other species such as spiders, springtails, which uses the carcass to build up their environment (Smith, 1986; Goff, 1993; Amendt, Krettek & Zehner, 2004). The first two categories consist of flies and beetles, which are major groups of insects attracted to carrion and may provide valuable information in forensic investigations (Amendt, Krettek & Zehner, 2004; De Souza, Kirst & Kruger, 2008).

According to Brundage (2020), insect species with unrestricted access to the carcass arrive and colonize the carcass within few minutes of animal’s death either to feed or colonize the carcass through oviposition. Anderson, Byrd & Castner (2001) reported that necrophagous insects from the families Calliphoridae and Sarcophagidae are primarily the first to colonize the body, and as the carcass decomposes, it subsequently attracts more insect species which colonizes the carcass in a sequential order until all valuable resources are depleted. Other experimental studies have shown that beetles arrive at a later stages of decomposition (Watson & Carlton, 2005; Wang et al., 2017; Matuszewski et al., 2020). Although insects typically develop at a predictable rate or sequence if the environmental conditions are suitable (Anderson, Byrd & Castner, 2001), their biological habits and feeding preferences differ (Hu et al., 2020).

The pattern of insect colonization mainly depends on the habitat, seasons, bio-geoclimatic zone, microclimate and the state of decomposition of the carcass (Anderson, 2000; Amendt, Krettek & Zehner, 2004; Joseph et al., 2011), and it is predictable within these parameters. This predictable time and sequence of arrival of insect on the carcass, allows forensic entomologists to determine the period or time of colonization (TOC) estimates, and consequently the time since death, also known as postmortem interval (PMI) (Smith, 1986; Anderson, Byrd & Castner, 2001; Benecke, 2001; Amendt et al., 2007; Brundage, 2020). The insects recovered from the carcass may also be used to determine the cause and manner of death, the transfer of the body between different locations after death, presence of drugs or poisons in a decomposing body and the linking of suspects with the crime/death scene in forensic investigations (Sukontason et al., 2007; Vanin et al., 2008; Joseph et al., 2011; Mise, Correa & Almeida, 2013). Therefore, correct identification of these arthropod species is a crucial element in forensic investigation (Chen, Hung & Shiao, 2004).

Arthropod species identification is mainly achieved through use of morphological and/or molecular techniques (Rolo et al., 2013). However, differentiation of some arthropod species especially cryptic species or larval stages using morphological approaches is still challenging (Amendt, Krettek & Zehner, 2004; Zehner et al., 2004; Boehme, Amendt & Zehner, 2012). Additionally, genetic analysis can accurately identify different arthropod species at all developmental stages (Oliveira et al., 2011; Kokdener, 2016). As a result, DNA methods have been widely used to identify arthropods species that are of importance in forensic investigations, consequently providing accurate PMI estimation (Park et al., 2013).

Previous research has illustrated the difference in arthropod species attracted by different animal models worldwide (Azwandi et al., 2013) ranging from pigs (Wolff et al., 2001; Kelly, Van der Linde & Anderson, 2011; Abajue, Ewuim & Akunne, 2017; Odo & Ilobo, 2020a), rabbits (De Souza, Kirst & Kruger, 2008; Shi et al., 2009; Mabika, Masendu & Mawera, 2014; Odo & Ilobo, 2020b), elephants (Coe, 1978) and rats (Moura, Carvalho & Monteiro-Filho, 1997; Carter, Yellowlees & Tibbett, 2008; Keshavarzi et al., 2019), however, limited studies have compared the assemblage and diversity of arthropod communities between different animal models (Watson & Carlton, 2003, 2005; Simmons, Adlam & Moffatt, 2010; Azwandi et al., 2013). Given the high rate of veterinary legal cases involving illegally killing or poaching of wildlife, this study aimed at identifying and comparing the diversity of arthropod communities between two animal models (pig and sheep) during different seasons in KwaZulu-Natal province of South Africa. This data will contribute to the existing carrion entomological database which can be used to solve local cases of suspicious wildlife death, illegally killing or poaching of wildlife in South Africa.

## Materials and Methods

### Ethics statement

This study contained animal subjects and complied with relevant ethical standards and was approved by the Animal Research Ethics Committee of the University of KwaZulu-Natal (AREC/048/018D) according to the South African national guidelines on animal care, handling and use in biomedical research.

### Study location

**Figure 1:**
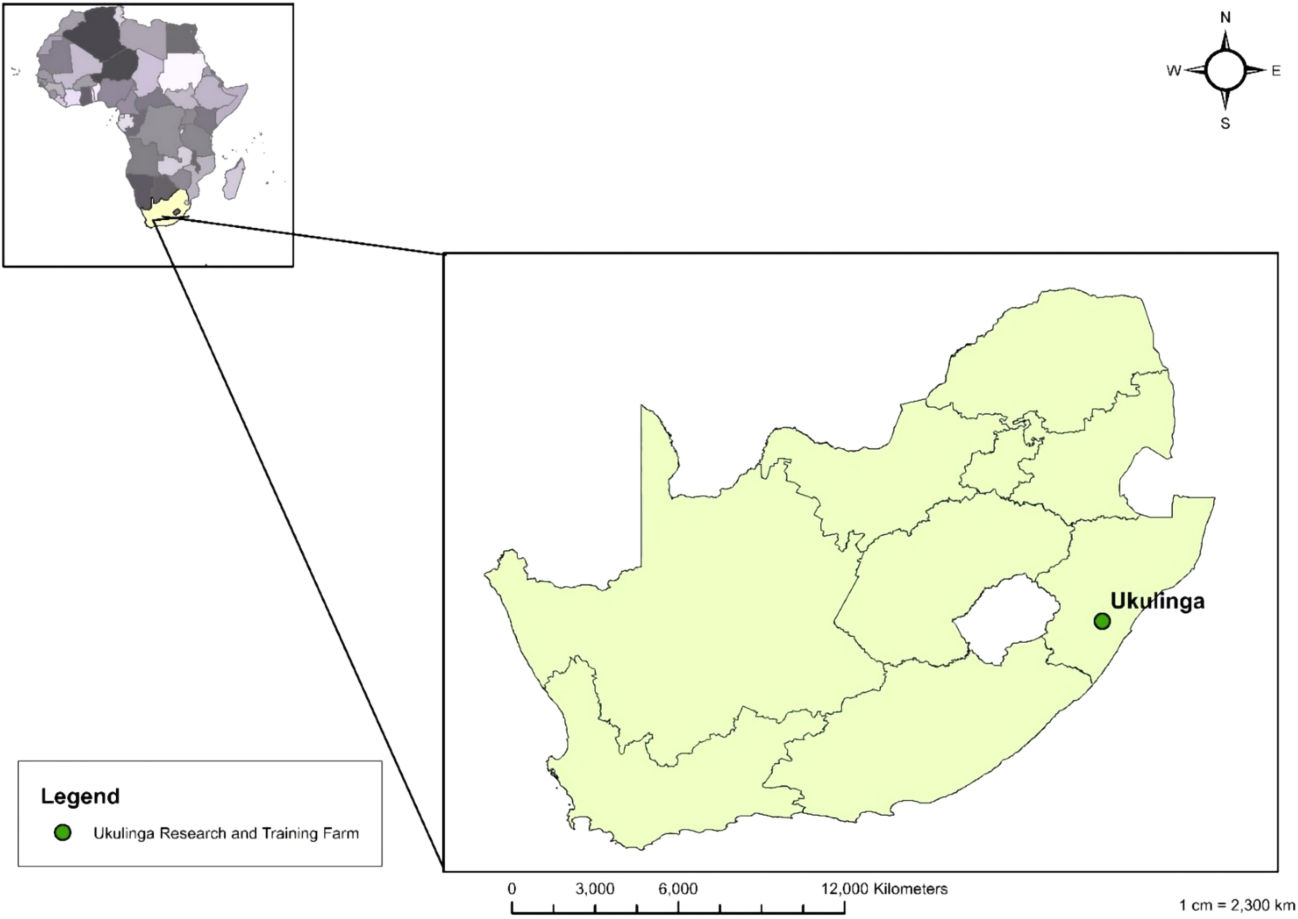
Map showing the study location in the KwaZulu-Natal province of South Africa (Tembe & Mukaratirwa, 2021).

Experimental trials were conducted at Ukulinga Research and Training farm located at the University of KwaZulu-Natal, Pietermaritzburg Campus, South Africa (Fig. 1). The study location is characterised by warm to hot summer, and mild winter temperatures which are usually accompanied by occasional frost (Kiala, Odindi & Mutanga, 2017). The area receives rainfall for approximately 106 days per year, with an average annual precipitation of 680 mm (Kiala, Odindi & Mutanga, 2017). The ecosystem of the farm is typically herbaceous due to long-term burning and it falls under the Southern Tall Grassveld (Mills & Fey, 2004; Kiala, Odindi & Mutanga, 2017). Experiments were performed during the cold season (June–August 2019) and warm season (November 2019–January 2020) with measured temperature ranging between 18–19 °C and 21–23 °C respectively (Tembe & Mukaratirwa, 2021).

### Study animals and sampling procedure

Two medium-sized healthy Merino sheep (*Ovis aries*) were donated by the University of KwaZulu-Natal Ukulinga Research and Training Farm and two pigs (*Sus scrofa domesticus*) were donated by Hmb School Trust piggery in Greytown, South Africa.

One pig and one sheep were used for the study in each season (cold and warm season). On day one of the study, experimental sheep were humanely killed by a stab in the heart after pre-slaughter stunning with a non-penetrating captive bolt and the donated fresh pig carcasses which died from sudden death with no signs of infection was immediately wounded by knife around the neck to mimic the case of illegally killed animal and placed in separate metal cages (100 cm × 100 cm × 100 cm) which were 200 m apart to minimize crossover of insects between carcasses. To prevent scavenging by predators including rats and other small vertebrates, cages were covered with mesh wire which still allowed free access of insects. Fly traps were hung on the cages to collect adult arthropods. Additional arthropods which were found on or around the carcasses were collected using forceps. Collection of arthropods were done for a period of two hours (9:00–11:00 am) daily. Arthropods were preserved in 70% ethanol prior transportation to the laboratory for morphological and molecular identification.

### Morphological identification

Adult arthropods were cleaned by soaking them in distilled water for 10–15 min and air dried (Tembe & Mukaratirwa, 2021). After drying, they were identified under a stereo microscope using identification keys based on body parts of the adult arthropods (Byrd & Castner, 2001; Kolver, 2009; Iqbal et al., 2014; Lutz et al., 2018; Lubbe et al., 2019; BugGuide, 2020).

### DNA extraction and amplification

Specimens were grouped into different genus based on the morphological identification. From each taxon two to four representative specimens were selected for confirmation of species using molecular techniques. Two or three legs (depending on size) of each dipteran fly specimens were collected carefully dissected out from the whole specimen and DNA was extracted using Genomic DNA™ Tissue Mini-Prep Kit (Zymo Research Cooperation) according to the manufacturer’s instructions. DNA were amplified using the universal mitochondrial primers LCO1490 (5′-GGTCAACAAATCATAAAGATATTGG-3′) and HCO2198 (5′-TAAACTTCAGGGTGACCAAAAAATCA-3′) (Folmer et al., 1994). PCR reactions were performed in a final volume of 25 μl containing the following PCR mixture: 2 μl of each primer (10 μM), 12.5 μl PCR Master Mix (2X) (Thermo Scientific), 4.5 μl sterile water and 4 μl of genomic DNA extract. Amplification was performed under thermal conditions: 95 °C for 7 min, followed by 35 cycles of (60 s at 95 °C, 60 s at 55 °C, 60 s at 72 °C) and final extension for 7 min at 72 °C.

For the coleopteran species, a separate PCR was performed to amplify the mitochondrial primers (F: 5′-CAGATCGAAATTTAAATACTTC-3′ and R: 5′-GTATCAACATCTATTCCTAC-3′) (Zhuang et al., 2011). PCR amplification was performed in a 25 µL reaction volume, each containing 5 µL of genomic DNA, 12.5 µL PCR Master Mix (2X) (Thermo Scientific), 2 µL (10 µM) of each primer and 3.5 µL sterile water under the thermal cycling conditions: 94 °C for 3 min, followed by 35 cycles of (94 °C for 30 s, 30 s at 50 °C, 30 s at 72 °C) and lastly final extension for 5 min at 72 °C. Fragments were separated by electrophoresis in 1% agarose gel stained with ethidium bromide, at 80V for 1 hour and amplifications were detected at 710 bp and 272 bp for the flies and beetles respectively. Amplicons were sent to Inqaba biotech industries (Pty) Ltd. (Pretoria, South Africa) for Sanger sequencing.

### Sequence and phylogenetic analysis

Sequences were assembled and manually edited using Bio Edit (Hall, 1999). Basic local alignment search tool (BLAST) of the NCBI (National Centre of biotechnology) was used to identify the closest matches available on the database. Sequences were aligned with the homologous sequences obtained from the GenBank databased using the MUSCLE option of MEGA 7 (Kumar, Stecher & Tamura, 2016). The sequences were trimmed to a common nucleotide length of 420 for the flies and 220 for beetles. The jModeltest (Posada, 2008) was used to determine the best model of nucleotide substitution to use in neighbor-joining, maximum likelihood and Bayesian inference analyses. The GTR+G model was selected for the datasets. To determine the evolutionary relationships between the arthropods, the Neighbor-joining (NJ) and maximum likelihood (ML) phylogeny trees were generated using PAUP*4.0 (Swofford, 2002), and the nodal support values were estimated using 1,000 bootstrap pseudo-replicated. Bayesian analyses were run using four Markov chains on MrBayes 3.1.2 (Huelsenbeck & Ronquist, 2001), sampling every 100 generations for 5 million generations or until the standard deviation of the split frequencies was less than 0.01. The first 500,000 trees were discarded as burn-in. The Bayesian inference phylograms were generated with 50% majority-rule consensus and the nodal support indicated as posterior probabilities.

### Statistical analysis

Chi-square test was used to test the influence of season on the abundance of carrion-associated arthropods groups using GraphPad. The difference was considered statistically significant if the *p*-value was *p* ≤ 0.05.

## Results

### Morphological identification of arthropod species

Morphological identification based on the identification key described by (Tourle, Downie & Villet, 2009; Iqbal et al., 2014; Lutz et al., 2018) classified collected dipteran flies into five genera; *Chrysomya*, *Lucilia*, *Musca*, *Sarcophaga* and one unidentified genus, and five Coleoptera families (Cleridae, Dermestidae, Silphidae, Scarabaeidae and Meloidae) (Tembe, Malatji & Mukaratirwa, 2021; Tembe & Mukaratirwa, 2021).

### Molecular identification of arthropod species

**Figure 2:**
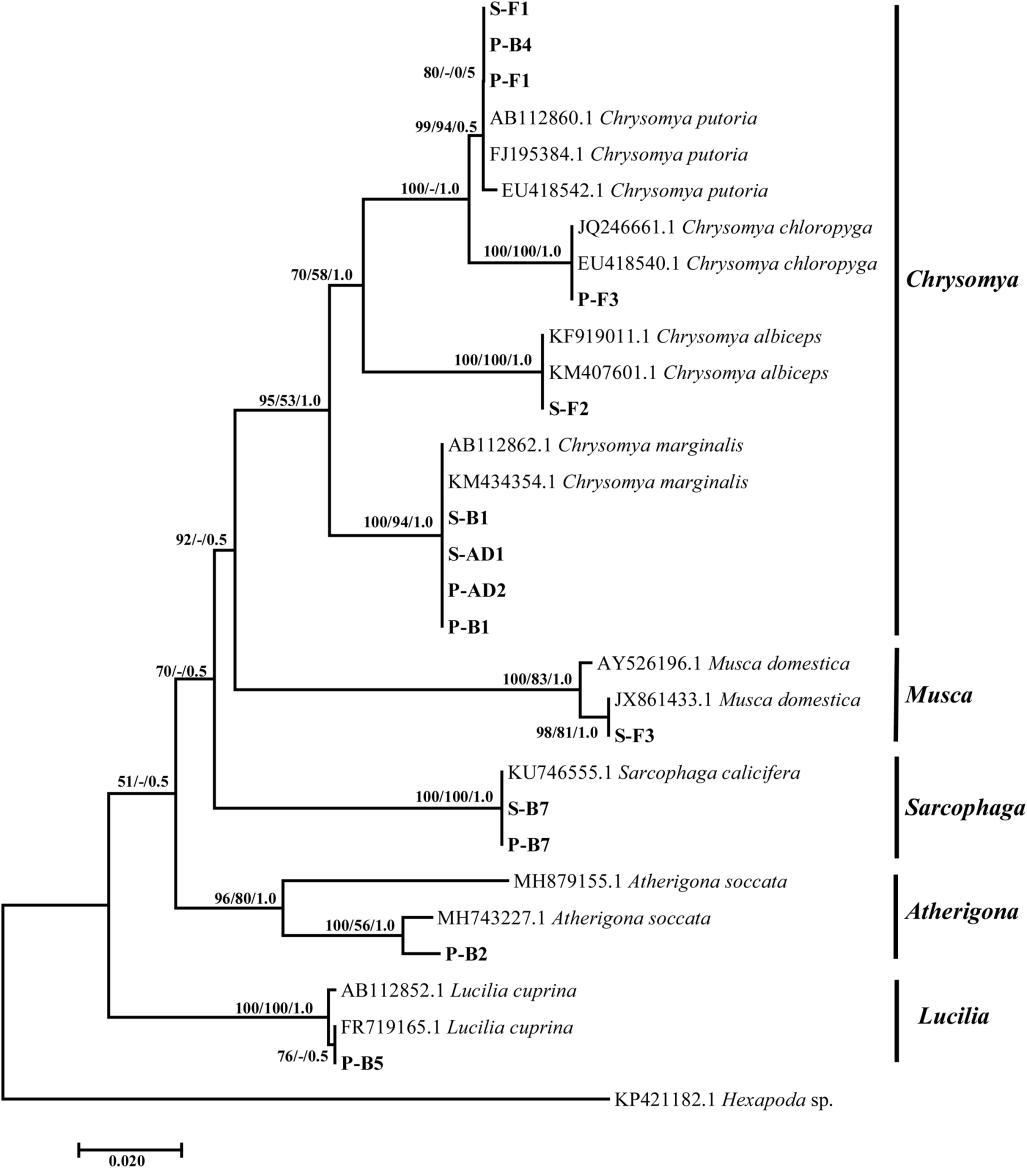
Tree based on the mitochondrial Cytochrome oxidase subunit one region illustrating relationships between experimental Diptera isolates collected from sheep and pig carcasses, and the close matches downloaded from the NCBI GenBank and outgroups. The sample ID alphabets represents: P-pig, S-sheep, F-fresh, B-bloated and AD-advanced stages. Support values indicated at the nodes are, in order: neighbor joining bootstrap value, maximum likelihood bootstrap value, Bayesian inference posterior probability.

Sequence analysis of the arthropods based on the mitochondrial gene confirmed morphological identification of the following blow fly species from the genera *Chrysomya Ch. marginalis* (Diptera: Calliphoridae) (Wiedemann, 1830); *Ch. putoria* (Diptera: Calliphoridae) (Wiedemann, 1880), *Ch. albiceps* (Diptera: Calliphoridae) (Wiedemann, 1819), *Ch. chloropyga* (Diptera: Calliphoridae) (Wiedemann, 1818), *Lucilia* (*L. cuprina*) (Diptera: Calliphoridae) (Wiedemann, 1830), *Musca* (*M. domestica*) (Diptera: Calliphoridae) (Walker, 1849). Molecular analysis further identified *Sarcophaga* and one unidentified genera up to species level (Fig. 2). Blast search showed that *Chrysomya* isolates from our study showed homologies of 100% with *Ch. marginalis* GenBank isolates (AB112862–South Africa, KM434354–Egypt), *Ch. putoria*, *Ch. albiceps* (JQ246661 and EU418540–South Africa) and *Ch. chloropyga* (KF919011–France, KM407601–Egypt). The sequences of isolates from our study were deposited in the GenBank under the accession numbers MZ476261–MZ476266, and MZ476272–MZ476274. *Lucilia cuprina* isolate (MZ476269) showed a homology of 99–100% with AB112852–Australia and FR719165–Kenya, with *M. domestica* (MZ476267) showing a homology of 99–100% to Brazilian isolate (AY526196) and South Korean (JX861433) respectively. The genera which was unidentified morphologically was identified as *Atherigona soccata* (MH743227, India), identity supported by a homology of 98%, and the specimen sequences were deposited under the accession number MZ476268. The *Sarcophaga* isolates (MZ476270–MZ476271) identified as *S. calicifera* with a 99% homology to a GenBank isolate from Burundi (KU746555).

Phylogeny analysis yielded a paraphyletic relationship between the dipteran species (Fig. 2). *Chrysomya* species formed their own well-supported clade by neighbor-joining and Bayesian inference showing the evolutionary relationship between the four *Chrysomya* species, and further supporting the identification of nine isolates from this study as *Ch. marginalis*, *Ch. putoria*, *Ch. albiceps* and *Ch. chloropyga*. The remaining four genera, namely *Lucilia*, *Musca*, *Sarcophaga* and *Atherigona* showed strongly supported clades with a close relationship within genera and species, and further authenticating the identification of the different species collected from the pig and sheep carcasses. However, these genera showed a weak to moderately supported paraphyletic relationship to one another. The unidentified genera (P-B2) formed a strong supported clade confirming the identification of this isolate as *A. soccata*, and this species was unique to the pig carcass during the bloated stage.

**Figure 3:**
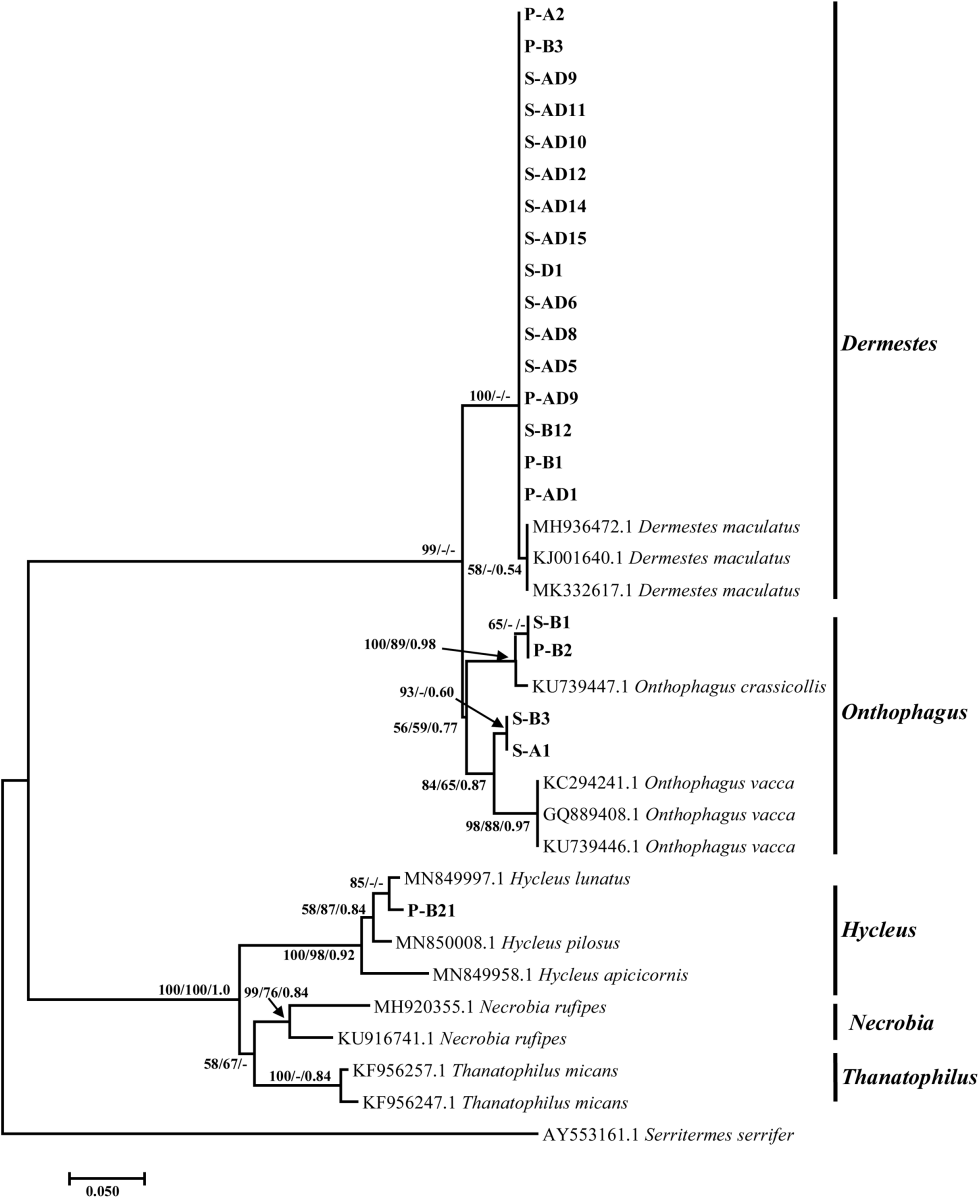
Tree based on the mitochondrial region illustrating relationships between experimental Coleoptera isolates collected from the sheep and pig carcasses, and the close matches downloaded from the NCBI GenBank and outgroups. The sample ID alphabets represents: P-pig, S-sheep, B-bloated, A-active, AD-advanced decay stage and D-dry stages. Support values indicated at the nodes are, in order: neighbor joining bootstrap value, maximum likelihood bootstrap value, Bayesian inference posterior probability.

Molecular analysis of the mitochondrial region of the beetles yielded sequences with a shorter nucleotide length. BLAST and phylogenetic analyses showed that 16 isolates from this study identified as *Dermestes maculatus* (Coleoptera: Dermestidae) (De Geer, 1774) with a percentage homology ranging from 98–99%. These isolates formed a monophyletic sister clade to *Onthophagus* species (Fig. 3), and their sequences were deposited into GenBank under the accession numbers MZ485937–MZ485952. Within the *Onthophagus* clade, two weakly supported monophyletic clades were formed supporting four isolates from this study as either *Onthophagus crassicollis* (Coleoptera: Scarabaeidae) (Boucomont, 1913) or *O. vacca*. Specimens identified as *O. crassicollis* were collected from both pig and sheep carcasses and showed homology of 98.68% with an isolate from the United Kingdom (KU739447) by BLAST analysis. The sequences were deposited into GenBank under the accession numbers MZ485955–MZ485956. *Onthophagus vacca* specimens from this study (MZ485953–MZ485954) showed a lower homology of 96.18% to the Australian isolate (KC294241), and this species was unique to sheep carcass. Lastly, isolate P-B21, which identified as *H. lunatus*, showed a homology of 98.48% to the Italian isolate (MN849997). This isolate (MZ485957) formed a well-supported clade by neighbor-joining method with the Italian isolate and a strong supported clade with other *Hycleus* species (Fig. 3).

### Arthropod diversity at different stages of a decomposing pig and sheep carcass during a cold season

Both sheep and pig carcasses were colonized by the same dipteran species during the first two stages of decomposition (fresh and bloated); namely *Ch. marginalis*, *Ch. putoria*, *Ch. albiceps*, *Ch. chloropyga*, *L. cuprina*, *M. domestica* and *Sarcophaga calcifera* (Diptera: Sarcophagidae) (Boettcher, 1912), plus *A. soccata* flies which were only found on the pig carcass during the bloated stage (Table 1). No beetles were collected on the pig carcass during these two stages of decomposition. However, *D. maculatus* and *O. vacca* appeared for the first time on the sheep carcass during the bloated stage, of which the *O. vacca* was specific to the sheep carcass.

**Table 1:** Arthropod diversity at different stages of a decomposing pig and sheep carcasses during cold season at a locality in KwaZulu-Natal province of South Africa.

Ecological category	Order	Family	Genus	Presence/absence of insects at different stages of pig and sheep carcass decomposition									
				Fresh (0–1 days)		Bloated (2–6 days)		Active (7–12 days)		Advanced (13–40 days)		Dry (41–50 days)	
				Pig	Sheep	Pig	Sheep	Pig	Sheep	Pig	Sheep	Pig	Sheep
Necrophagous	Diptera	Calliphoridae	*Chrysomya marginalis*	**+**	**+**	**+**	**+**	**+**	**+**	**+**	**+**	**-**	**-**
Necrophagous	Diptera	Calliphoridae	*Chrysomya putoria*	**+**	**+**	**+**	**+**	**-**	**+**	**-**	**+**	**-**	**-**
Necrophagous	Diptera	Calliphoridae	*Chrysomya albiceps*	**+**	**+**	**+**	**+**	**+**	**+**	**+**	**+**	**-**	**-**
Necrophagous	Diptera	Calliphoridae	*Chrysomya chloropyga*	**+**	**+**	**+**	**+**	**+**	**+**	**-**	**-**	**-**	**-**
Necrophagous	Diptera	Calliphoridae	*Lucilia cuprina*	**+**	**+**	**+**	**+**	**+**	**+**	**-**	**+**	**-**	**-**
Necrophagous	Diptera	Muscidae	*Musca domestica*	**+**	**+**	**+**	**+**	**+**	**+**	**+**	**+**	**+**	**+**
Necrophagous	Diptera	Muscidae	*Atherigona soccata*	**-**	**-**	**+**	**-**	**-**	**-**	**-**	**-**	**-**	**-**
Necrophagous	Diptera	Sarcophagidae	*Sarcophaga calicifera*	**+**	**+**	**+**	**+**	**+**	**+**	**+**	**+**	**+**	**+**
Predator	Coleoptera	Cleridae	*Necrobia rufipes*	**-**	**-**	**-**	**-**	**+**	**+**	**+**	**+**	**+**	**+**
Necrophagous	Coleoptera	Dermestidae	*Dermestes maculatus*	**-**	**-**	**-**	**+**	**+**	**+**	**+**	**+**	**+**	**+**
Predator	Coleoptera	Silphidae	*Thanatophilus micans*	**-**	**-**	**-**	**-**	**-**	**-**	**+**	**+**	**-**	**-**
Coprophagous	Coleoptera	Scarabaeidae	*Onthophagus vacca*	**-**	**-**	**-**	**+**	**-**	**+**	**-**	**-**	**-**	**-**
Coprophagous	Coleoptera	Scarabaeidae	*Onthophagus crassicollis*	**-**	**-**	**-**	**-**	**-**	**+**	**+**	**+**	**+**	**+**

**Note:**

+, presence; -, absence.

### Arthropod diversity at different stages of a decomposing pig and sheep carcass during a warm season

Both pig and sheep carcasses were colonized by the same dipteran species (Table 2). Similar beetle species colonized both sheep and pig carcasses with exception to *O. vacca* which was found only on the sheep carcass. However, there were differences in time of arrival of beetles between pig and sheep carcasses. *Dermestes maculatus*, *T. micans* and *O. crassicollis* beetles were found on the sheep carcass during the fresh stage, however, colonization of same beetle species and *H. lunatus* on the pig carcass commenced during the bloated stage.

**Table 2:** Arthropod diversity at different stages of a decomposing pig and sheep carcasses during warm season at a locality in KwaZulu-Natal province of South Africa.

Ecological category	Order	Family	Genus	Presence/absence of insects at different stages of pig and sheep carcass decomposition									
				Fresh (0–1 days)		Bloated (2–6 days)		Active (7–12 days)		Advanced (13–40 days)		Dry (41–50 days)	
				Pig	Sheep	Pig	Sheep	Pig	Sheep	Pig	Sheep	Pig	Sheep
Necrophagous	Diptera	Calliphoridae	*Chrysomya marginalis*	**+**	**+**	**+**	**+**	**+**	**+**	**+**	**+**	**-**	**+**
Necrophagous	Diptera	Calliphoridae	*Chrysomya putoria*	**+**	**+**	**+**	**+**	**-**	**+**	**-**	***+***	**-**	**-**
Necrophagous	Diptera	Calliphoridae	*Chrysomya albiceps*	**+**	**+**	**+**	**+**	**+**	**+**	**+**	**+**	**-**	**-**
Necrophagous	Diptera	Calliphoridae	*Chrysomya chloropyga*	**+**	**+**	**+**	**+**	**+**	**+**	**+**	**+**	**-**	**-**
Necrophagous	Diptera	Calliphoridae	*Lucilia cuprina*	**+**	**+**	**+**	**+**	**+**	**+**	**-**	**-**	**-**	**-**
Necrophagous	Diptera	Muscidae	*Musca domestica*	**+**	**+**	**+**	**+**	**+**	**+**	**+**	**+**	**+**	**+**
Necrophagous	Diptera	Sarcophagidae	*Sarcophaga calicifera*	**+**	**+**	**+**	**+**	**+**	**+**	**+**	**+**	**+**	**-**
Predator	Coleoptera	Cleridae	*Necrobia rufipes*	**-**	**-**	**-**	**-**	**+**	**+**	**+**	**+**	**+**	**+**
Necrophagous	Coleoptera	Dermestidae	*Dermestes maculatus*	**-**	**+**	**+**	**+**	**+**	**+**	**+**	**+**	**+**	**+**
Predator	Coleoptera	Silphidae	*Thanatophilus micans*	**-**	**+**	**+**	**+**	**+**	**+**	**+**	**+**	**+**	**+**
Coprophagous	Coleoptera	Scarabaeidae	*Onthophagus vacca*	**-**	**-**	**-**	**+**	**-**	**+**	**-**	**+**	**-**	**-**
Coprophagous	Coleoptera	Scarabaeidae	*Onthophagus crassicollis*	**-**	**+**	**+**	**+**	**+**	**+**	**+**	**+**	**+**	**+**
Incidental	Coleoptera	Meloidae	*Hycleus lunatus*	**-**	**-**	**+**	**+**	**-**	**-**	**-**	**-**	**-**	**-**

**Note:**

+, presence; -, absence.

### Arthropod species richness from pig and sheep carcass during cold and warm seasons

The overall number of arthropods species collected from the pig and sheep carcasses were equal (*n* = 12) during the cold season (Table 1). Whilst, more (*n* = 13) arthropod species were collected from the sheep carcass during the warm season (Table 2). The highest number of arthropod species were found during the bloated stage of the pig carcass (*n* = 11) and both bloated and active decomposition stage of sheep carcass (*n* = 12) in warm season (Table 2). The overall number of Diptera species found on the pig carcass during the cold season was eight (8) whilst only seven (7) species were found on the sheep carcass (Table 1). The difference in the number of dipteran species found between the two animal models was due to the presence of *A. soccata*, which was unique to the bloated stage of pig carcass during cold season only. Although the pig carcass generally attracted more dipteran species, reduction in the number of these species on the carcass started as early as the advanced stage whilst most species left the sheep carcass mainly during the dry stage. The sheep carcass presented five coleopteran species, which was more than the four found on the pig carcass during the cold season. The difference is attributed by the presence of *O. vacca* which was unique to the sheep carcass during the active and advanced stages of decomposition.

Both carcasses attracted the same amount of dipteran species (*n* = 7) during the warm season (Table 2). As with the cold season, the pig carcass was the first animal model which presented an early reduction of dipteran species which started as early as the active stage with the disappearance of *Ch. putoria*. Seven coleopteran species were found on the sheep carcass whilst pig presented only six species. The difference was contributed by the presence of *Onthophagus* sp. which was also unique to the sheep carcass during the warm season as well. The results also showed the presence of *H. lunatus*, which was unique to the bloated stage of both sheep and pig carcasses only during the warm season.

### Arthropod abundance from pig and sheep carcass during cold and warm seasons

There was a significant relationship (*p* < 0.01) between species of Diptera with the animal model, indicating that the number of dipterans were dependent on the pig and sheep model during the cold and warm season and with coleopterans the dependence was only during the warm season. Overall dipterans were more abundant (*i.e.*, more flies were collected on and around the pig carcass) during the warm (*n* = 1,519) and cold (*n* = 779) seasons as compared to sheep carcass during the warm (*n* = 511) and cold (*n* = 229) season (Table 3). Coleopterans were more abundant on the sheep carcass during the warm (*n* = 391) and cold (*n* = 135) seasons as compared to the pig carcass in both warm (*n* = 261) and cold (*n* = 114) seasons (Table 3). Generally, more flies and beetles were collected on both sheep and pig carcasses during the warm season as compared to the cold season (n = 1,272) (Table 3).

**Table 3:** Summary of seasonal abundance of carrion-associated insect species collected pig and sheep during both cold and warm season.

Order	Family	Species	Cold season		Warm season	
			Pig	Sheep	Pig	Sheep
Diptera	Calliphoridae	*Chrysomya marginalis*	210	47	356	103
Diptera	Calliphoridae	*Chrysomya putoria*	87	23	202	44
Diptera	Calliphoridae	*Chrysomya albiceps*	150	50	303	110
Diptera	Calliphoridae	*Chrysomya chloropyga*	91	15	276	37
Diptera	Calliphoridae	*Lucilia cuprina*	38	17	60	46
Diptera	Muscidae	*Musca domestica*	191	71	298	110
Diptera	Muscidae	*Atherigona soccata*	2	0	0	0
Diptera	Sarcophagidae	*Sarcophaga calicifera*	10	21	24	61
Total Diptera			779	244	1,519	511
Chi-square			47.0773, *p* < 0.0001		156.5752, *p* < 0.0001	
Coleoptera	Cleridae	*Necrobia rufipes*	37	36	59	53
Coleoptera	Dermestidae	*Dermestes maculatus*	66	68	112	146
Coleoptera	Silphidae	*Thanatophilus micans*	2	4	44	72
Coleoptera	Scarabaeidae	*Onthophagus vacca*	0	5	0	22
Coleoptera	Scarabaeidae	*Onthophagus crassicollis*	9	22	42	92
Coleoptera	Meloidae	*Hycleus lunatus*	0	0	4	6
Total Coleoptera			114	135	261	391
Chi-square			10.3056, *p* = 0.067		27.8024, *p* < 0.0001	
Total (Diptera and Coleoptera)			893	379	1,780	902

*Chrysomya* spp. and *M. domestica* were dominant on the pig carcass on both seasons, contributing more than 90% of the total number of flies collected on and around the pig carcass during the warm and cold season respectively. The sheep carcass was mainly dominated by *Ch. marginalis*, *Ch. albiceps* and *M. domestica* which contributed more than half of the total number of flies collected on and around the carcass. *Dermestes maculatus* and *N. rufipes* were the most dominant beetle species on both pig and sheep carcasses during the cold season. These two species contributed about 89% and 77% of the total number of beetles collected on the sheep and pig carcasses respectively. The same species were dominant on the pig carcass during the warm season, and these two beetle species contributed 65.5% of the total beetles collected. However, the sheep carcass was dominated more by *D. maculatus*, *N. rufipes*, *T. micans* and *O. vacca* which was unique to the sheep carcass. These species were dominant on the sheep carcass, making up 79% of the beetles collected on the sheep carcass during the warm season.

## Discussion

Morphological identification of flies, supported by molecular analysis based on the mitochondrial gene identified *Ch. marginalis; Ch. putoria, Ch. albiceps, Ch. chloropyga*, *L. cuprina*, *M. domestica*, *S. calcifera* and *A. soccata* collected from various stages of pig and sheep carcasses. After several unsuccessful attempts to amplify the beetles with various mitochondrial and nuclear markers in our study, amplification was achieved using primers as described by Zhuang et al. (2011). However, molecular analysis showed low resolution of the beetle specimens due to the short nucleotide sequence size obtained and failure to obtain usable sequences for specimens identified morphologically such as *T. micans* and *N. rufipes* (Byrd & Castner, 2001; Kolver, 2009; BugGuide, 2020).

Previous studies showed that arthropod species composition varies per carcass type/animal model (Watson & Carlton, 2003; Azwandi et al., 2013). Watson & Carlton (2003) observed differences in arthropod species composition between black bear, white-tailed deer, alligator and swine. Similar observations were made by Azwandi et al. (2013) who reported differences in number of arthropod species obtained in rat (*n* = 47), rabbit (*n* = 55) and monkey (*n* = 54). Combined morphological and molecular techniques in this study also showed a difference in arthropod species observed during the warm season whereby the sheep carcass attracted more arthropod species (*n* = 13) compared to the pig carcass (*n* = 12). This difference in the number of arthropod species from these two animal models was observed to be attributed to the presence of beetle species *O. vacca* which was unique to the sheep carcass.

In contrast, there were no observed variation in the number of insect species (*n* = 12) collected and identified on pig and sheep carcasses during the cold season. Although the number of insect species collected from these carcasses were similar, 11 of the 12 species collected were common to both pig and sheep carcasses, with two insect species observed to vary per animal type. This includes a Muscidae species, *A. soccata* which was only collected on the pig carcass during the cold season, and a Scarabaeidae species *O. vacca* which was only collected on the sheep carcass during both cold and warm seasons. Similar observation was made by Watson & Carlton (2003) although on different animal species, who documented 11 arthropod families which were present on the black bear, white-tailed deer and swine carcasses, however, absent from the alligator carcass. Considering that both sheep and pig carcasses shared the same ecological niche, it can be concluded that these two arthropod species are unique to their respective animal models because every animal model has its own physical characteristics that are not only limited to the difference in size but also affected by factors such as the diet of the animal and the thickness of their furs among other factors (Azwandi et al., 2013).

Studies have showed that insects become attracted to the carcass immediately after death (Nuorteva, 1977; Erzinclioglu, 1983; Smith, 1986; Anderson & VanLaerhoven, 1996; Dillon, 1997). According to Byrd & Castner (2001), calliphorids are often the primary colonizers and are attracted to a carcass from a long distance by odor, and in South Africa, this is marked by flies of the genus *Chrysomya* (Braack, 1981). The same observations were made in this study where both sheep and pig carcasses, regardless of the season were assembled by blow flies from the genus *Chrysomya* and *Lucilia* along with the flesh flies (*Sarcophaga*) and house fly (*Musca*) within the first stage of decomposition. This observation is in line with Byrd & Castner (2001) report that species from the families Calliphoridae (*Chrysomya* and *Lucilia*), Muscidae (Musca) and Sarcophagidae (*Sarcophaga*) are the most encountered species on carrions and serve as most useful evidence in forensic investigations. However, their species often vary depending on factors such as geographic region or bio-geoclimatic zones (Byrd & Castner, 2001).

There was an observed difference in the arrival time of the Coleoptera species communities between animal models and seasons. Assemblage of beetles were observed to begin earlier on the sheep carcass as compared to the pig carcass, and during the warm season as compared to the cold season. During the cold season, the first coleopteran species were observed and collected on the sheep carcass during the bloated stage and the active stage on the pig carcass. In the cold season, the highest number of coleopteran species were collected during the active (*n* = 4) and advanced (*n* = 4) stages for sheep carcass and during the advanced stage (*n* = 4) on the pig carcass. During the warm season, beetles were collected from the sheep carcass as early as the fresh stages, whilst on the pig carcass observation and collection of beetles only began during the bloated stage. The highest number of beetle species were on both sheep (*n* = 5) and pig (*n* = 4) carcasses observed during the bloated, active decay and advanced decay stages.

More dipterans were collected from the pig carcass during both warm and cold seasons, as compared to the sheep carcass. In contrast, sheep carcasses attracted more coleopterans during both warm and cold as compared to the pig carcasses in warm and cold seasons. According to Azwandi et al. (2013), the difference in assemblages of arthropod communities between animal models may be due to the amount food provided by the animal type, for instance, the pig carcasses in this study, the body/food was exposed and easily accessible compared to the sheep carcasses which was covered by the wool as a result, pig carcasses provided more food in terms of body tissue and fluids to the necrophagous species.

According to Byrd & Castner (2001), the ability of certain insects to colonize a carcass during a particular season and their relative abundance can provide valuable information such as determining the season of death. In this study, coleopteran species *H. lunatus* was collected on the sheep and pig carcasses during the warm season only. Furthermore, the dipteran species *A. soccata* was collected on the pig carcass during the cold season. These species may potentially serve as valuable indicators for determining season of death, and the animal species with regards to *A. soccata*. However, the lower number of individuals collected may also indicate the high probability of accidental colonization.

## Conclusions

The results showed minimal arthropod diversity between pig and sheep carcasses. Season had an influence on collected arthropod species richness and abundance where the overall number of arthropods collected from the two animal models during the warm season were high compared to cold season. The observed difference in arthropod abundance between seasons in this study is likely to have been influenced by temperature. These results contribute new knowledge on the seasonal diversity of arthropod species in two animal models in KwaZulu-Natal province of South Africa, which will furnish the increasing accuracy in determining PMI in forensic investigation cases such as illegal poaching in this region. We recommend further studies on arthropod composition and diversity of different animal species in different parts of South Africa.

## Additional Information and Declarations

### Data Availability

The following information was supplied regarding data availability: The sequences of the studied Diptera and Coleoptera specimens are available at GenBank: MZ485937–MZ485957.
